# Effect of Redox Conditions on Bacterial Community Structure in Baltic Sea Sediments with Contrasting Phosphorus Fluxes

**DOI:** 10.1371/journal.pone.0092401

**Published:** 2014-03-25

**Authors:** Anne K. Steenbergh, Paul L. E. Bodelier, Caroline P. Slomp, Hendrikus J. Laanbroek

**Affiliations:** 1 Netherlands Institute of Ecology (NIOO-KNAW), Wageningen, The Netherlands; 2 Department of Earth Sciences (Geochemistry), Faculty of Geosciences, Utrecht University, Utrecht, The Netherlands; 3 Institute of Environmental Biology, Science Faculty, Utrecht University, Utrecht, The Netherlands; CSIR- National institute of oceanography, India

## Abstract

Phosphorus release from sediments can exacerbate the effect of eutrophication in coastal marine ecosystems. The flux of phosphorus from marine sediments to the overlying water is highly dependent on the redox conditions at the sediment-water interface. Bacteria are key players in the biological processes that release or retain phosphorus in marine sediments. To gain more insight in the role of bacteria in phosphorus release from sediments, we assessed the effect of redox conditions on the structure of bacterial communities. To do so, we incubated surface sediments from four sampling sites in the Baltic Sea under oxic and anoxic conditions and analyzed the fingerprints of the bacterial community structures in these incubations and the original sediments. This paper describes the effects of redox conditions, sampling station, and sample type (DNA, RNA, or whole-cell sample) on bacterial community structure in sediments. Redox conditions explained only 5% of the variance in community structure, and bacterial communities from contrasting redox conditions showed considerable overlap. We conclude that benthic bacterial communities cannot be classified as being typical for oxic or anoxic conditions based on community structure fingerprints. Our results suggest that the overall structure of the benthic bacterial community has only a limited impact on benthic phosphate fluxes in the Baltic Sea.

## Introduction

Due to eutrophication coastal ecosystems are increasingly under threat by the spreading of so-called dead zones, in which oxygen concentrations fall below a level necessary for higher life [1]. Because phosphorus (P) release from sediments is enhanced under low oxygen conditions, a positive feedback loop between eutrophication, low oxygen concentrations and P release from sediments can occur [2]–[4]. Various studies have shown that there is an intricate relation between microbial processes and benthic P release, with microbes contributing both to enhanced release and retention of P in sediments [5]–[7]. The influence of microbes on P release is potentially sensitive to the absence or presence of oxygen, as this determines which metabolic pathways can be used by bacterial communities. Previous studies have shown correlations between redox conditions and prokaryote community structure in coastal Baltic Sea sediments [8], [9]. However, in observational studies it is often difficult to isolate the effect of redox condition on community structure form other, often co-varying, environmental variables.

In the present study we used controlled experiments to investigate the influence of redox condition on bacterial community structure. This study is part of a larger project in which the effects of microorganisms on benthic phosphorus (P) cycling are investigated. This paper describes the effect of oxic and anoxic conditions on the bacterial community structure of Baltic Sea sediment. The results are discussed in the light of P dynamics in these sediments.

## Methods

### Sediment Sampling and Incubation

**Figure 1:**
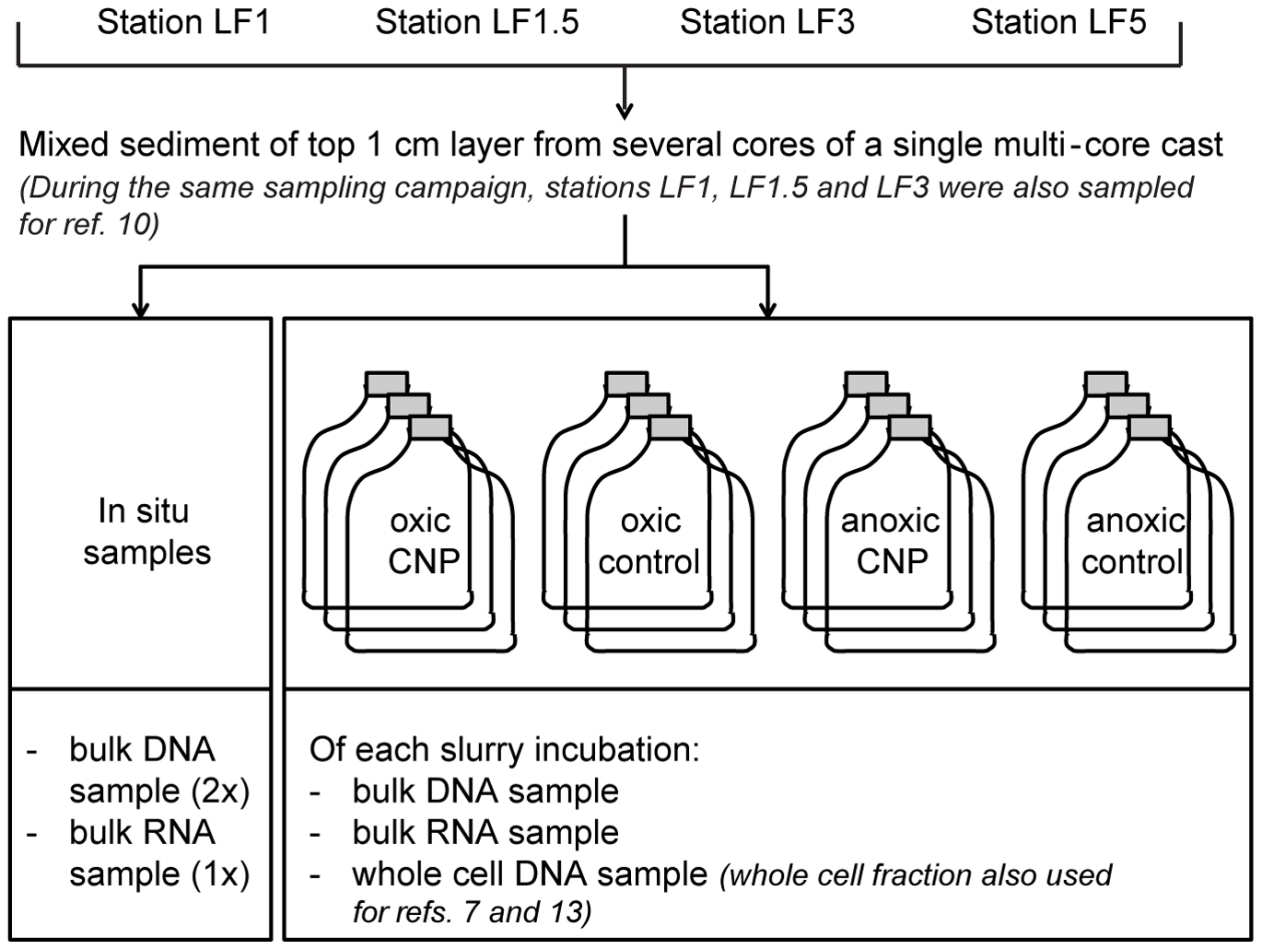
Schematic overview of samples and replicates.

An overview of the sampling- and incubation-scheme is given in Figure 1. The top layers (0–1 cm) of recently deposited, organic matter-rich sediments and bottom water were sampled at four stations in the Baltic Sea. The sampling stations (LF1; LF1.5; LF3; LF5) are located along a depth transect from oxic to anoxic bottom water conditions in the Baltic Sea. The R/V Aranda had permission to sample these study sites for scientific purposes (granted by the Estonian and Swedish coast guard). A summary of site and sediment characteristics is given in Table S1 in File S1; for a more extensive description see [10]. Although the sediments at these four stations are similar in their general geochemical composition (e.g. organic carbon and total aluminum contents), their P chemistry is different due to their position relative to the oxycline [10]. The bottom water redox conditions at the stations range from oxic at station LF1 to anoxic and sulfidic at station LF5 as a result of the increasing water depth (from 67 to 135 m) along the approximately 70 km long sampling transect. Sediment from several cores within a multi-core cast was combined in air-tight glass containers, bottom water was filter sterilized (0.22 μm) into sterile bottles, and both transported back to the laboratory at approximately 4°C in the dark.

Back in the laboratory, the sediments were well mixed and subsampled under constant nitrogen flushing in a glove bag (Glas-Col, Terre Haute). Approximately 5 g of sediment with 12.5 mL of bottom water was weighed into 50 mL serum bottles. To reduce pH changes during incubation, HEPES buffer was added to the bottom water (4-(2-hydroxyethyl)-1-piperazineethanesulfonic acid, pH 7.4, final concentration 25 mM). The slurries were flushed during 1 h with nitrogen (for the anoxic incubations) or compressed air (oxic incubations). The sediment slurries were incubated on a gyratory shaker (∼125 rpm) in the dark at 5.2°C, which is the average temperature of the bottom water across the four stations at the time of sampling. Two treatments were given in triplicate to the incubations in a full factorial design: redox condition (either oxic or anoxic) and carbon-and-nutrient amendment (either control [no amendment] or CNP [carbon, nitrogen and phosphorus] amendment), resulting in 48 incubations (4 stations × 2 redox conditions × 2 amendments × 3 replicates). The CNP amendment was given to ensure the availability of carbon and nutrients for bacterial growth under the imposed redox conditions. The CNP-amended slurries received glucose, Sørensen’s phosphate buffer (pH 7.4) and NH_4_Cl (10, 1, and 1 mmol L^−1^ final concentration, respectively). To reduce the build up of CO_2_ in the incubations and to prevent oxygen depletion in the oxic incubations, after the first two weeks of the incubation time the incubation bottles were flushed weekly with either nitrogen or compressed air.

At the end of the incubation period (77–86 days, which allows for on average 4 cell division cycles when a specific growth rate of 0.05 d^−1^ is assumed at this temperature [11]), subsamples were taken for extraction of bulk DNA, bulk RNA, and DNA from the whole-cell fraction. For the latter a subsample of sediment slurry was blended, followed by centrifugation with a Nycodenz (Axis-Shield PoC AS, Oslo, Norway) density-cushion on which bacteria are concentrated following the method of Lindahl [12]. The whole-cell fraction was also used in two accompanying studies in which the percentages of phosphatase-expressing bacteria were determined by flow-cytometry [7], and cellular C:N:P ratios were determined by X-Ray Microanalysis [13]. More details regarding sediment sampling, incubation, and separation of the whole-cell fractions is published elsewhere as part of an accompanying study focusing on the role of phosphatase activity in the sediments [7].

### Molecular Analysis of the Bacterial Community Structures

We assessed the structure of the active bacterial community (“RNA”; analysis of ribosomal RNA), the total bacterial community (“bulk DNA”; analysis of 16S rRNA genes), and whole-celled bacterial community (“whole cell” analysis of 16S rRNA genes of density-separated whole cells). The whole-cell community includes inactive cells that are not detected on basis of RNA, but excludes extracellular DNA and DNA present in lysed cells. The community structures were analyzed by Denaturing Gradient Gel Electrophoresis (DGGE). The DGGE banding patterns were analyzed by Functional PCA (FPCA) [14]. FPCA compares the entire intensity profile of each lane to the full dataset, thereby taking the location of each intensity peak (i.e. DGGE band) into account. In this way FPCA analysis uses more of the information contained in the DGGE profiles compared to standard DGGE analysis, for which each DGGE lane profile is divided into unrelated bins. The phylogenetic affiliation of selected DGGE bands was used to describe the bacterial communities on phylum- and class-level.

#### Bulk DNA extraction from sediment

All tubes used for nucleic acid extraction and PCR reactions were exposed to UV light on a UV trans-illuminator for approximately 20 minutes, to prevent DNA contamination. DNA was extracted as described by Steenbergh et al. [15], with minor modifications. Approximately 0.5 g of freeze-dried non-incubated sediment, or 1 ml of freeze-dried sediment slurry was used for DNA extraction. Upon extraction, DNA pellets were resuspended in 50–75 μl of PCR-grade water. The DNA concentration was determined by spectrophotometry (NanoDrop® ND-1000; Nanodrop Technology, Wilmington, DE). The bulk DNA extractions were diluted to 10 ng μl^−1^ prior to PCR amplification.

#### Bulk RNA extraction from sediment

At the end of the slurry incubations, 2 ml slurry subsamples for RNA extraction were centrifuged (16.1×g for 15 min) in 2 ml screw-cap tubes. The supernatant was removed, 300 μl of RNA*later* (Ambion) was added and the sediment pellet resuspended. Samples were kept at 4°C for one week, after which they were centrifuged (16.1×g for 15 min) and the RNA*later* was removed with a syringe and needle. For RNA extraction of the non-incubated sediment, approximately 0.5 g of fresh sediment was weighed into a 2 ml screw-cap tube to which an approximate equal volume of RNA*later* was added and mixed. Samples were stored at 4°C for one week, after which they were centrifuged (16.1×g for 1 min) and the RNA*later* was pipetted off. All RNA*later*-treated samples were stored at −20°C until RNA extraction. To each sample, 1 ml of CTAB+ buffer [16] and approximately 0.5 g of zirconia/silica beads (0.1 mm; Biospec Products Inc.) was added. A FastPrep-24 bead beater (MP Biomedicals, Solon, OH) was used to disrupt the cells (6 ms^−1^ for 45 s), after which the samples were cooled on ice. Proteinase K (5 μl of 20 mg ml^−1^) was added and samples were incubated for 30 min at 37°C with mixing before and halfway during the incubation. SDS (150 μl, 20% w/v) was added, followed by 1 hour incubation at 65°C with mixing before and every 20 minutes during the incubation. Samples were centrifuged (18890×g for 5 min) and 600 μl of the supernatant transferred to a new tube. Sediment pellets were re-extracted by adding 450 μl of CTAB+ and 50 μl of the SDS solution, mixing and incubating for 10 min at 65°C. An additional 400 μl of the supernatant after centrifugation (18890×g for 5 min) was added to the previously collected supernatant. Phenol/CIA (1 ml phenol : chloroform : isoamylalcohol, 25∶ 24 : 1, v/v/v) was added to the samples and mixed. After centrifugation (21500×g for 5 min) 750 μl of the supernatant was transferred to a new tube, to which 750 μl of CIA (24∶1, v/v) was added and mixed. Samples were centrifuged (21 500×g for 5 min) and 720 μl of the supernatant transferred to a new tube. Nucleic acids were precipitated by adding 750 μl of PEG solution (20% w/v polyethylene glycol 8000, 2.5 M NaCl in sterile RNase-free water) and incubating for 1 hour at 37°C. The supernatant was removed after centrifugation (21 500×g for 5 min at 4°C) and 500 μl of ice-cold ethanol (70% w/v in RNase-free water) was added to clean the pellet. To remove ethanol, samples were centrifuged (18890×g for 5 min), the supernatant removed and pellets dried in a Speed Vac (DNA Speed Vac DNA 110, Savant). The pellets were resuspended in 40 μl of RNase-free water. The nucleic acid concentration was determined as described above and diluted to 50 ng μl^−1^ prior to DNase treatment with the TURBO DNA-*free*™ Kit (Ambion). To 10 μl of the dilution 1.15 μl of 10×TURBO DNase buffer and 0.5 μl of TURBO DNase was added, mixed and incubated for 30 min at 37°C. Another 0.5 μl of TURBO DNase was added, followed by a second incubation step (30 min at 37°C). The reaction was stopped by addition of 2 μl of DNase inactivation agent, 5 min incubation at room temperature and centrifugation (10000×g for 1.5 min). The supernatant containing the RNA was transferred to a new tube. The complete removal of amplifiable DNA was confirmed by the absence of PCR products when performing a PCR-amplification as described below on the RNA samples prior to the reverse transcription step.

#### DNA extraction from whole-cell fraction

To prevent contamination of the low quantities of DNA in the whole-cell fractions with foreign DNA (for example from the reagents), a different method was used for the DNA extraction from the whole-cell fraction compared to the bulk DNA extractions. After freeze drying of the sorted material, 100 μl of PCR-grade water was added to each sample. DNA was released from the cells by three cycles of snap-freezing in liquid nitrogen (1 min) and subsequent thawing. To reduce the salt content of the samples, 10 μl of the extract was dialyzed for 15 min using membrane filters (Millipore MF 0.025μm VSWP) floating on approximately 25 ml of MilliQ water in a petri dish. The dialyzed DNA extracts were transferred to new tubes.

#### RT-PCR

cDNA was synthesized using SuperScript III Reverse Transcriptase (Invitrogen), according to the manufacturer’s instructions. To each reaction 4 μl of diluted DNase-treated sample (50 ng μl^−1^) and 1 μl RNase-free water was added.

#### PCR amplification

Primers used for direct PCR amplification in combination with DGGE analysis were universal eubacterial primers F357 to which a GC-clamp was attached (GC-F357; 5′-CGC CCG CCG CGC CCC GCG CCC GGC CCG CCG CCC CCG CCC CCC TAC GGG AGG CAG CAG-3′) and R518 (5′-ATT ACC GCG GCT GCT GG-3′). For nested PCR amplification of the whole-cell fractions primers F27 (5′-AGA GTT TGA TYM TGG CTC AG-3′) and 1492R (5′- TAC GGY TAC CTT GTT ACG ACT-3′) were used in the first step (all primers: Thermo Fisher Scientific, Ulm, Germany).

PCR reactions volumes were 25 μl (unless stated otherwise), containing 12.5 μl 2×PCR premixture (Epicentre Technologies, Madison, WI), 2.5 μl of 5 pM of both forward and reverse primer, 0.125 μl of recombinant *Taq* polymerase (Invitrogen), and 1 μl of (c)DNA sample. All PCR amplifications were performed in an MBS 0.5 S thermocycler (ThermoHybaid, Ashford, UK). The thermal-cycle profile was based on the touchdown profile described by Muyzer and colleagues [17], with the following modifications. The *Taq* polymerase was added already before the start of the PCR amplification, and the profile was extended by adding 10 extra cycles at 55°C annealing temperature (making 35 cycles in total). The primer extension step at the end of the profile was 5 min. at 72°C. For the reamplification of cut DGGE bands the profile was shortened by 10–14 cycles at the end of the profile.

#### PCR of whole-cell samples

To increase the sensitivity of the PCR amplification, a nested approach was used for the DNA from the whole-cell fraction. In the first step of the nested PCR, primers F27 (5′-AGA GTT TGA TYM TGG CTC AG-3′) and 1492R (5′-TAC GGY TAC CTT GTT ACG ACT-3′) were used (Thermo Fisher Scientific, Ulm, Germany). The thermal cycle profile was the standard profile as described above. For the second step, primers GC-F357 and R518 (see above) were used and the thermal cycle profile was reduced to the first 30 cycles.

#### DGGE

Bromophenol loading buffer was added to the PCR products and DGGE performed with a 20–80% denaturing gradient in 8% acrylamide gels (where 100% denaturant is 7 M urea and 40% formamide) in a Protean II system (Bio-Rad, USA) at 60°C, 100 V for 17 h. Gels were stained with ethidium bromide and the banding patterns recorded (ImaGo compact imaging system, B&L Systems, The Netherlands). The software package Phoretics 1D (version 2003.02; Nonlinear Dynamics) was used to analyze the banding patterns by removing background intensity from each lane, assigning standardized retardation factors (Rf) and determining the relative intensity of each band to the total intensity in each lane. DGGE bands selected for sequencing were excised and reamplified until single DGGE bands were obtained. Bands that could not be reamplified were manually excluded from analysis (e.g. heteroduplexes). Purification and sequencing (forward and reverse) of the PCR products was performed by Macrogen (Korea).

#### DGGE analysis

**Figure 2:**
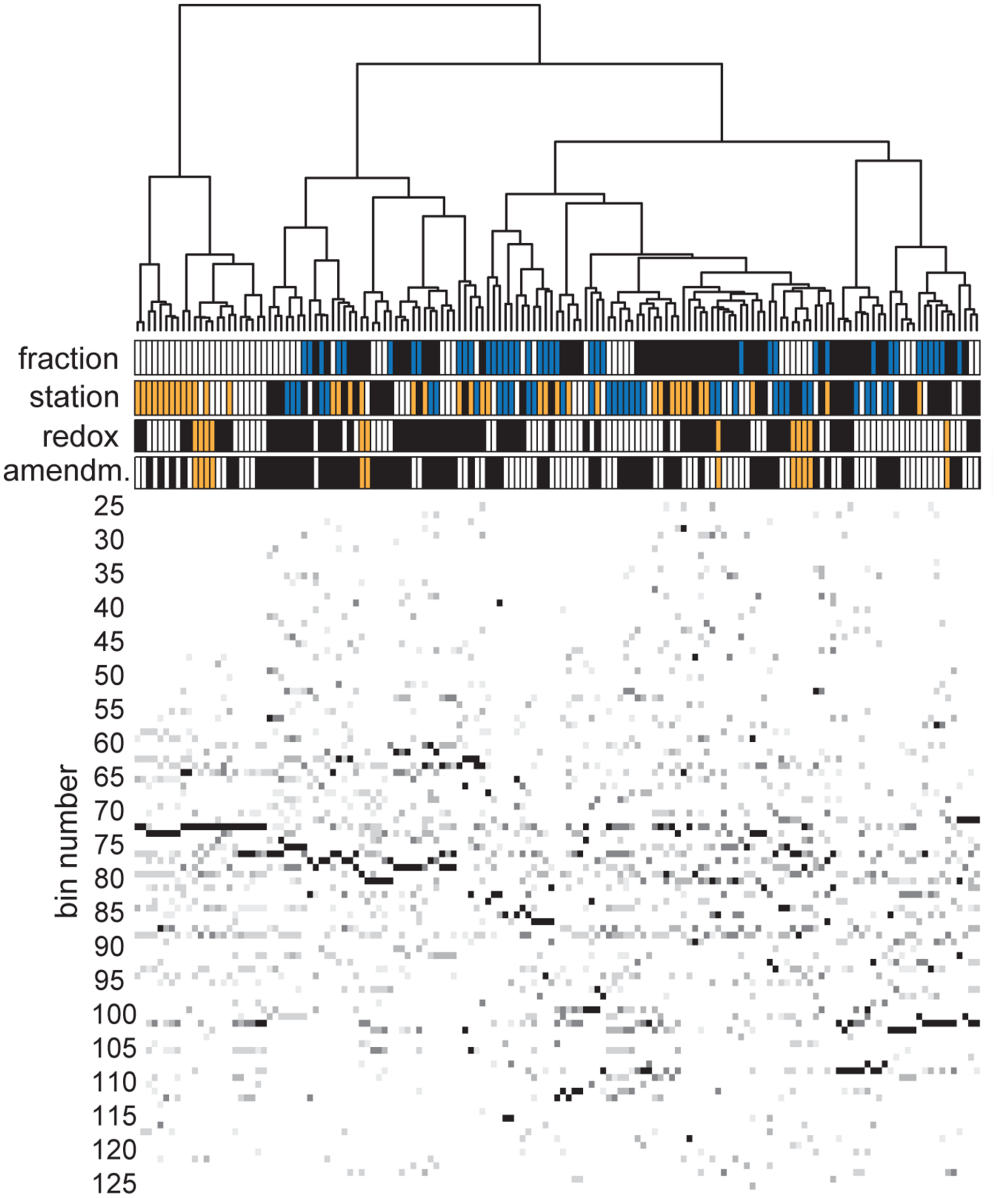
Visual representation of the DGGE fingerprints. Each vertical line of pixels represents the DGGE lane of a single sample. Lower bin numbers correspond to the top of the DGGE gel. Higher relative intensities of DGGE bins are represented with darker pixels. The clustering dendrogram shows the relation between samples. The sample fraction, sampling station, amendment and redox condition of each sample is color- coded between the dendrogram and the fingerprints (fraction: bulk DNA = white, whole-cell DNA = blue, RNA = black; station: LF1 = white, LF1.5 = yellow, LF3 = blue, LF5 = black; amendment: control = white, CNP = black, in situ = yellow; redox: oxic = white, anoxic = black, in situ = yellow).

The digitized DGGE lane profiles were cropped to cover the same section of the gel, and divided into 101 equally sized bins (see Figure 2). The intensity of each of these bins relative to the total lane intensity was determined.

#### DGGE analysis by functional PCA (FPCA)

The DGGE intensity profiles of each sample were used for FPCA [14]. Because the software package used for DGGE analysis (Phoretics 1D) does not have the option to export information on the intensity profiles corrected for nonlinearity with the use of DGGE markers, the intensity profiles were manually aligned with the peaks assigned by Phoretics (because the peaks are corrected for nonlinearity). The aligned intensity profiles were corrected with R-function smooth.spline. The corrected intensity profiles were used for functional PCA using R-function “pca.fd” from R package fda [18].

### Community Structure Analysis

The distance matrix based on the FPCA scores was calculated with R-function “dist” using Euclidean distances. Ward hierarchical clustering dendrograms were constructed based on the distance matrix with R-function “hclust”. For Correspondence Analysis (CA) the R-function “cca” (package vegan) [19] was used on the distance matrix of FPCA scores. Correlation between the abundance of individual DGGE bins and total community structure was determined with R-function “envfit” (package vegan).

#### PERMANOVA

To describe how the variation in the community structure based on FPCA scores is attributed to the different factors (i.e., station, redox, amendment, and sample-fraction) we used R-function “adonis” with Euclidean distances (Permutational Multivariate Analysis of Variance Using Distance Matrices, PERMANOVA; package vegan).

### Phylogenetic Analysis

The forward and reverse sequences of selected DGGE bands were aligned using the software package Sequencher 4.2 (Gene Codes Corporation, Ann Arbor, MI). The resulting contigs were visually inspected and ambiguities manually resolved. Low-quality contigs were removed from the dataset. Aligning and filtering were performed using mothur version 1.19.3 [20]. Phylogenetic tags were assigned to the sequences based on the RDP (Ribosomal Database Project) naïve Bayesian rRNA Classifier (Version 2.2, March 2010) [21]. Sequences with a lower than 95% score at phylum level were left unclassified (“unknown”).

The phylogenetic tag of a sequence was given to all bins of the same height, not only to the lane from which the original band was cut for sequencing. Exceptions to this were when bins received more than one phylogenetic tag. In this case, bins were either labeled with only one tag which differed across stations (when bins received tags that were consistent within a station, but different across stations), or labeled with a double tag (when lanes from the same station had received different tags, or when no tag was available for the bin at one station while the same bin received more than one different tags at other stations). Bins for which no phylogenetic tag was available were labeled “unknown”. To calculate the abundance of each phylogenetic group, the relative intensities of the DGGE bins that were classified as belonging to this group were summed for each DGGE lane. Analysis of variance of the phylogenetic community structure across stations and environmental variables was performed using R-function “aov”.

## Results and Discussion

The total dataset used for community structure analysis consists of 147 samples: 56 bulk DNA samples (4 duplicate in situ DNA samples and one DNA sample of each of the 16 triplicate slurry incubations), 52 RNA samples (4 in situ samples and one RNA sample of each of the 16 triplicate slurry incubations), and 39 whole-cell fraction DNA samples (one sample of 1–3 of the 16 triplicate slurry incubations; not all PCR reactions of this fraction gave a PCR product; see Figure 1).

The digitized DGGE lane profiles of this dataset were analyzed by FPCA. Figure 2 shows a representation of these DGGE lane profiles. Each vertical line of pixels represents the DGGE profile of a single sample. The color coding above the DGGE pattern shows the combinations of variables that belong to each sample, as specified in the figure legend. The cluster dendrogram at the top of the figure shows the similarity between the samples. From this dendrogram it can be seen that the bacterial communities from the sediment before incubation (the in-situ samples; yellow symbols in the color-coding row of ‘redox’ and ‘amendment’) do not form a cluster separate from the communities of the incubated samples. Therefore, even though the bacterial communities have changed during incubation, the observed variation in community structure in the incubations broadly falls within the range that is observed for the in situ samples.

From Figure 2 it is also clear that the samples do not cluster consistently according to either sample fraction (bulk DNA, whole-cell DNA, or RNA), station, redox condition (oxic or anoxic), or amendment (control or CNP).

**Figure 3:**
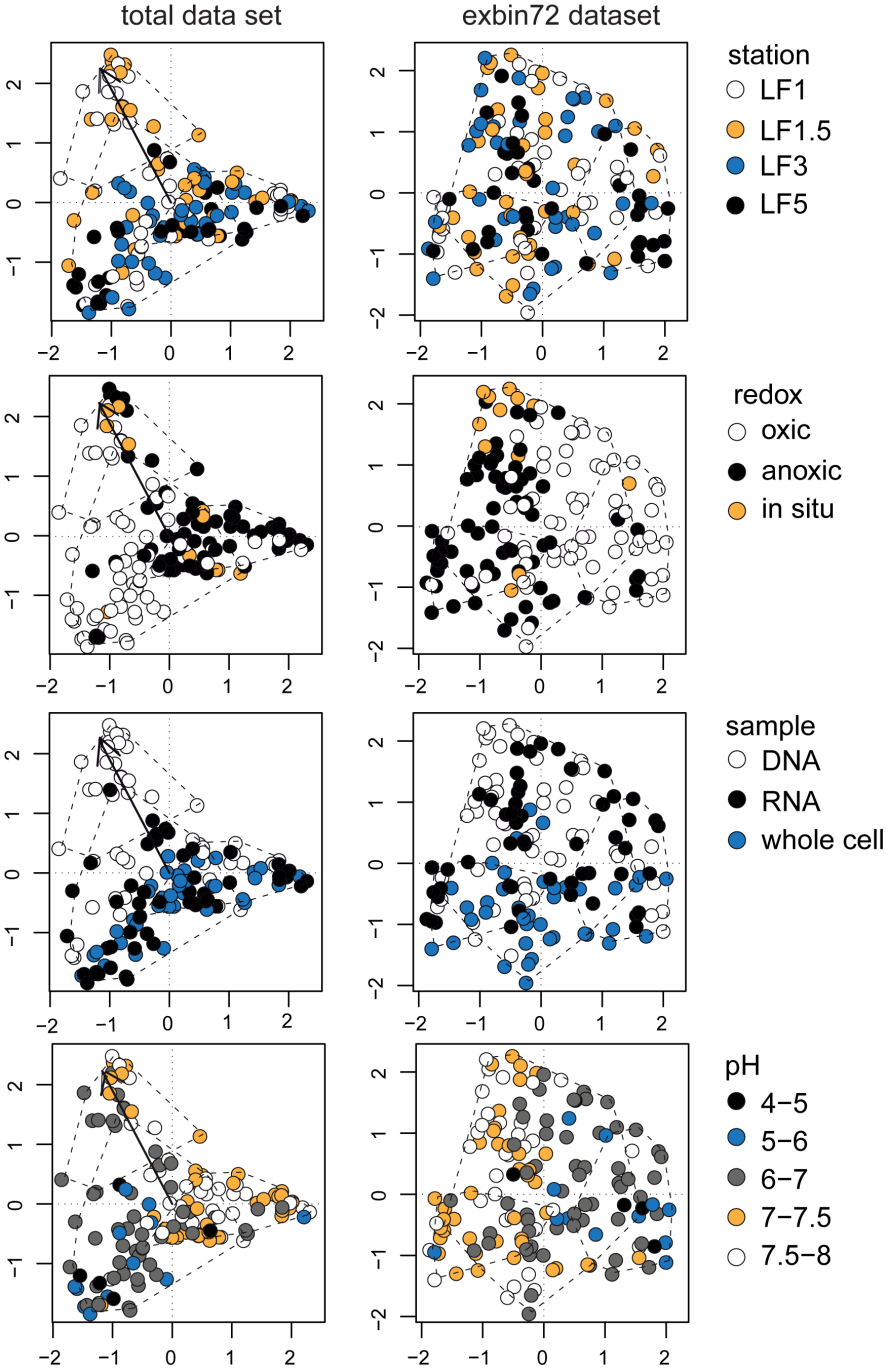
Correspondence Analysis plot based on Functional PCA of DGGE fingerprints. The left-hand panels represent the original dataset. The percentage of total variance in this dataset explained by axis 1 and 2 is 22.3% and 20.4%, respectively. The right-hand panels represent the exbin72 dataset (in which the abundance of bin 72, assumed to be of cyanobacterial origin, has been set to zero; see Results section); the percentage of variance explained by the axes is 28.2% and 15.1%. The symbols in the replicate plots are coded according to the legends. The dashed lines represent the three major clusters of the clustering dendrograms (see Figure 1), and the solid arrow in the left-hand panels the fit of bin 72 to community structure (r^2^ = 0.58; see text and Table S2 in File S1). The pH of the incubations was determined at the end of the incubation period. The in situ pH of station LF3 was not determined, and is assumed to be 7.5.

Another way of visualizing the difference between the community structure of the sediment samples is a Correspondence Analysis (CA) plot (see Figure 3). Again, the samples do not form clearly defined clusters in ordination space (Figure 3, left-hand panels). The three main clusters of the dendrogram in Figure 2 are represented by dashed lines in the CA plot. The cluster of samples in the top left-hand corner corresponds to the left cluster of the dendrogram in Figure 2 and consists mainly of bulk DNA samples from stations LF1 and LF1.5.

The proportion of variance in the community structure that is explained by the different variables can be determined with PERMANOVA (see Table 1). The variance between replicate samples accounts for 36% of the total variance in the dataset. The factor explaining most of the variance (8%) is sample fraction (bulk DNA, whole-cell DNA, or RNA fraction).

**Table 1:** Permutational Analysis of Variance (PERMANOVA) of the original dataset and the exbin72 dataset.

		original dataset			exbin72 dataset		
	Df	F	R^2^	P	F	R^2^	P
fraction	2	9.4633	0.076	***	5.2885	0.047	***
station	3	4.8702	0.058	***	2.705	0.036	***
redox	2	6.2903	0.050	***	6.5052	0.058	***
amendment	1	6.4723	0.026	***	6.1723	0.028	***
fraction × station	6	2.6613	0.064	***	1.6314	0.044	**
fraction × redox	3	2.2346	0.027	***	2.2419	0.030	***
station × redox	6	2.2344	0.054	***	2.0401	0.055	***
fraction × amendment	2	2.613	0.021	***	2.1697	0.019	***
station × amendment	3	3.0225	0.036	***	3.0524	0.041	***
redox × amendment	1	4.8778	0.019	***	4.5292	0.020	***
fraction × station × redox	9	2.2194	0.080	***	2.0007	0.080	***
fraction × station × amendment	6	1.7111	0.041	***	1.6316	0.044	***
fraction × redox × amendment	2	2.3632	0.019	***	2.2247	0.020	***
station × redox × amendment	3	2.4228	0.029	***	2.3909	0.032	***
fraction × station × redox × amendment	6	1.5329	0.037	***	1.5171	0.041	**
Residuals	91		0.364			0.406	
Total	146						

Significance codes: *** = 0.001, ** = 0.001<0.01.

Exbin72 dataset = dataset in which the relative intensity of DGGE bin 72 has been excluded from the FPCA analysis (see Results section).

### Effect of Cyanobacterial DNA on Community Structure

**Figure 4:**
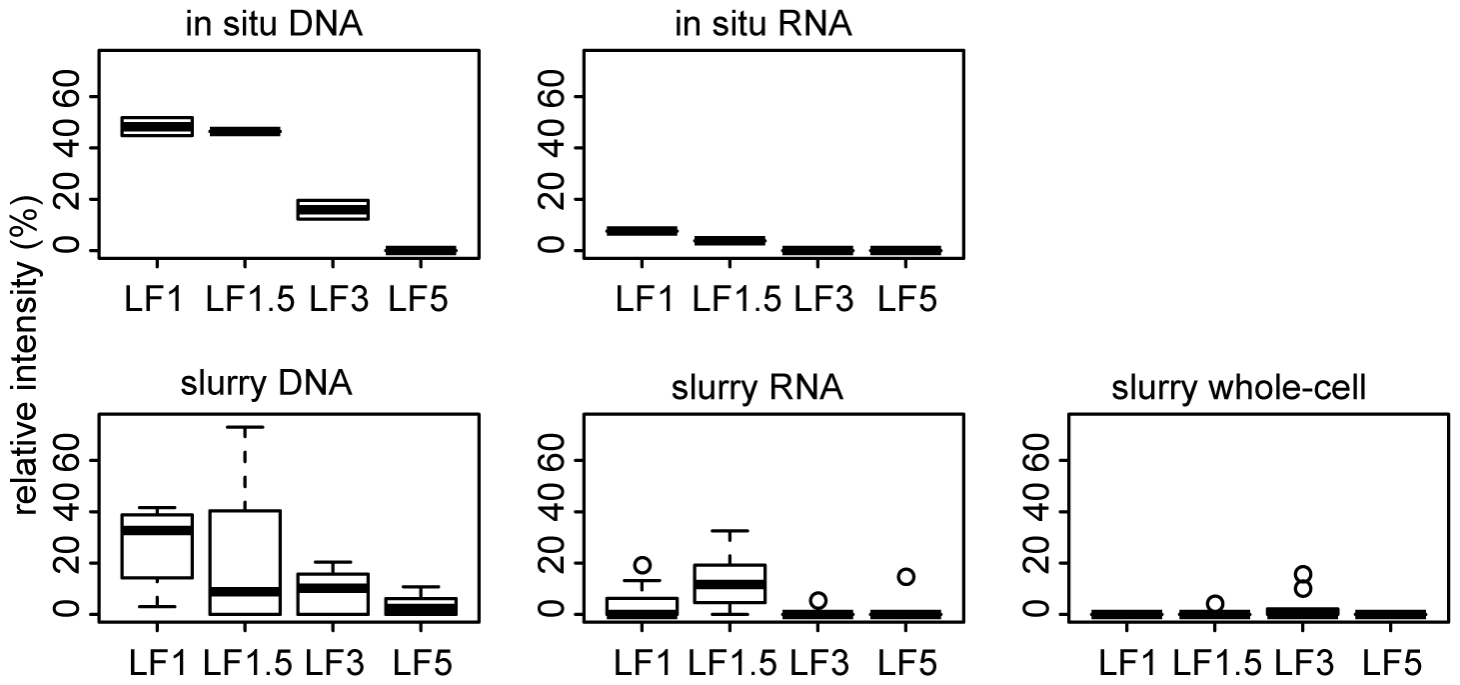
Relative intensity of DGGE bin 72. The relative intensity of DGGE bin 72 of bulk DNA, whole-cell, and RNA fraction of in situ and incubated samples.

To determine the relation between specific DGGE bands and the total community structure, the digitized DGGE profiles were subdivided into 101 bins per lane (as seen in Figure 2). The relative abundance of each bin was fitted to the CA. Bins with a significant correlation to the total community structure are given in Table S2 in File S1. The relative abundance of bin 72 has the best fit with community structure (r^2^ = 0.58). Figure 4 shows the relative abundance of this bin across stations and sample types. While bin 72 is highly abundant in the bulk DNA fraction of the in situ samples from station LF1 and LF1.5, it is far less abundant in the RNA fraction. The overall abundance of bin 72 is lower in the incubated samples, but the trend across stations and DNA/RNA fractions is similar to the in situ samples, with exception of the relatively high abundance in the RNA fraction of the incubations of station LF1.5. The abundance of bin 72 in the whole-cell DNA fractions of the incubated samples is close to zero.

Bands from bin 72 have been excised from 4 separate lanes and were classified as *Cyanobacteria* (3 bands) and *Proteobacteria* (1 band). On the basis of this classification and the lower abundance in RNA and whole-cell DNA fractions it is likely that the high relative abundance of this DGGE band at station LF1 and LF1.5 is a remnant of settled plankton from the water column. It has been shown that settling of cyanobacterial blooms from the water column can indeed lead to a high abundance of cyanobacterial DNA in sediment top layers in the Baltic Sea [22].

Although the high relative abundance of the 16S rRNA genes of what presumably are dead cyanobacteria at stations LF1 and LF1.5 is not an artifact, it may preclude underlying community structures as the shape of the FPCA harmonics is influenced by the large intensity peaks at the position of this bin. To exclude the effect of bin 72 on the community structure, a new dataset was made for FPCA in which the abundances of the section of each lane corresponding to the region of bin 72 (approximately bins 71 to 74) were set to 0. This dataset will be referred to as “exbin72”. In this exbin72 dataset the variance between replicates accounts for 41% of the total variance, and redox condition is the factor explaining most of the variance (6%; PERMANOVA, see Table 1). Due to the removal of the influence of bin 72 from the dataset, the data points corresponding to the DNA fraction of stations LF1 and LF1.5 no longer form a separate cluster in ordination space (Figure 3, right-hand panels).

### Effect of Redox Condition on Community Structure

When the influence of cyanobacterial abundance is removed from the dataset (i.e. in the exbin72 dataset), the redox condition is the factor explaining most of the variance between samples. However, redox condition explains only 6% of the variance (PERMANOVA; see Table 1) and there is a large overlap between communities from oxic and anoxic incubations in ordination space (see ‘redox’ in Figure 3, right-hand panel). This contrasts with previous studies where oxic and anoxic bacterial communities were compared using 16S rRNA gene fingerprinting techniques; for example bacterial communities from vertical redox gradients in sediments in the Baltic Sea were clearly separated according to the redox potential [8]. Also a study in which tropical soil was incubated under different redox conditions, showed a clear distinction between oxic and anoxic communities [23]. In contrast to these studies, in the present study a nested experimental design was used with additional variables next to redox condition: sampling station, amendment, and sample fractions (bulk DNA, whole-cell DNA and RNA fraction).

**Figure 5:**
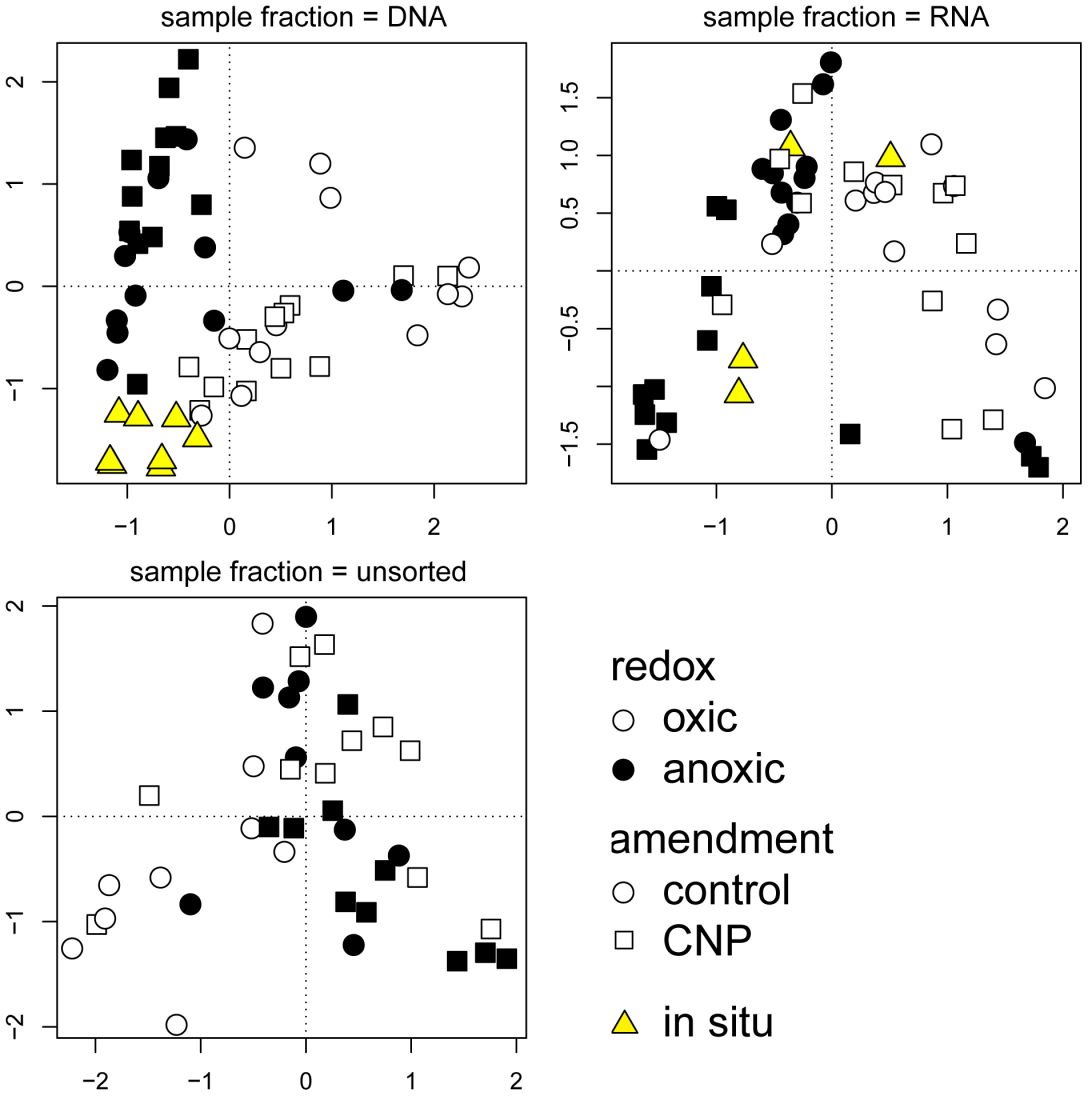
Correspondence Analysis plots based on Functional PCA of DGGE fingerprints of the exbin72 dataset of the DNA, RNA, and whole-cell fraction. The percentage of total variance in this dataset explained by axis 1 and 2 is 28.6% and 17.1% for the DNA fraction, 42.3% and 16.1% for the RNA fraction, and 21.2% and 15.3% for the whole-cell fraction. The shape and fill of the symbols in the subplots are coded according to the legends.

To separate the influence of the sample fraction on bacterial community structure from the other variables, we have constructed separate datasets for the three sample fractions. These three datasets are subsets of the exbin72 dataset. For each sample fraction, the functional principal components were calculated and used for further analysis. The CA plots of the separate sample fractions are shown in Figure 5. For the DNA fraction the community structures from oxic and anoxic incubations are more separated. The variation in redox condition is mainly directed along the first axis of the CA plot, which is the axis that explains most variation. On this axis, the in situ DNA samples (yellow symbols in the plot) are closer to the anoxic slurry incubations. As the top centimeter of sediment was used and the maximal oxygen penetration depth at the stations was 1.5 mm (see table S1 in File S1), most of the in situ sediment will have experienced anoxic conditions.

Interestingly, the three anoxic samples that cluster together with the oxic samples are the three replicates of the non-amended incubations of station LF5, which is the station with anoxic and euxinic conditions at the time of sampling.

In contrast to the DNA fraction, the community structures of oxic and anoxic conditions of both RNA and whole-cell fraction of the slurry incubations overlap. This suggests that the active bacterial communities under oxic and anoxic conditions are more similar to each other than the structure of the total bacterial community (including non-active and dead bacteria, as determined by DNA analysis). The active bacterial communities of the two sampling stations with oxic bottom water (stations LF1 and LF1.5; the two lower ‘in situ’-symbols in the RNA plot of Figure 5) cluster more closely with the active communities of anoxic incubations than with those of oxic incubations.

The proportion of variation in community structure that can be explained by redox condition was determined by PERMANOVA (see Table 2). The explained proportion was larger for the DNA fraction of the incubations (14%) than for the RNA and whole-cell fractions (11% and 5%, respectively).

**Table 2:** Permutational Analysis of Variance (PERMANOVA) of the separate exbin72 datasets of the bulk DNA, whole-cell DNA, and RNA fraction.

	DNA fraction				RNA fraction				whole-cell fraction			
	Df	F	R^2^	P	Df	F	R^2^	P	Df	F	R^2^	P
station	3	4.50	0.12	***	3	1.77	0.07	**	3	1.01	0.07	
redox	2	8.09	0.14	***	2	4.34	0.11	***	1	2.12	0.05	**
amendment	1	4.62	0.04	***	1	4.16	0.05	***	1	2.41	0.05	**
station × redox	6	2.98	0.16	***	6	2.96	0.22	***	3	1.20	0.08	
station × amendment	3	3.32	0.09	***	3	1.82	0.07	**	3	1.48	0.10	*
redox × amendment	1	4.55	0.04	***	1	3.82	0.05	***	1	1.72	0.04	*
station × redox × amendment	3	2.99	0.08	***	3	1.39	0.05	.	3	1.31	0.09	.
residuals	36		0.32		32		0.39		23		0.52	
total	55		1.00		51		1.00		38		1.00	

Significance codes: *** = 0.001, ** = 0.001<0.01, * = 0.01<0.05.

In conclusion, while subsets of our data display a separation between oxic and anoxic samples (e.g., the DNA fraction of the incubations), this distinction between oxic and anoxic communities does not hold for the entire dataset and also not for the RNA and whole-cell fraction of the incubations. This overlap in patterns between bacterial communities from oxic and anoxic incubation conditions can be caused by several reasons:

1. Many members of the community are facultative aerobes/anaerobes and therefore can occur under both oxic and anoxic conditions.
2. Rare members of the total community may be responsible for the major biogeochemical reactions. Differences in the relative abundance of these rare members would go unnoticed in our DGGE-based approach. Uncoupling of community composition based on DGGE-fingerprints and major enzymatic reactions has been shown for stream biofilm communities [24].
3. Bacterial community structure is determined by many variables simultaneously, which results in a lack of a consistent pattern in the way that bacterial communities from different sampling locations are affected by redox condition during incubation.
4. The size of the bacterial seed bank [25]. In case the seed bank of inactive bacterial species is large it may mask changes in the small proportion of active cells. Although the analysis of 16S rRNA is generally used to assess active communities (as we do in this study), 16S rRNA content does not scale directly with microbial activity; dormant cells can even have higher 16S rRNA contents than actively growing cells [26]. Members of the bacterial seed bank can therefore be detected within all three sample fractions (DNA, RNA and whole-cell fraction).
5. In natural settings, redox condition can influence bacterial community structure indirectly, as sediments overlain by oxic or anoxic bottom waters will experience different degrees of bioturbation. Bioturbation not only affects the transport of oxygen into the sediment, but for example also distribution of labile organic matter [27]. As our incubations were continuously shaken, the effect of bioturbation on bacterial community structure was excluded in our setup.

### Effect of Other Factors on Community Structure

The bacterial community structures of the full dataset also overlap in ordination space across the three sample fractions (bulk DNA, whole-cell, and RNA fractions, see Figure 3). Depending on whether or not the effect of cyanobacterial abundance is excluded from the data, the factor sample fraction accounts for 8% (complete dataset) and 5% (exbin72 dataset) of the variance in community structure (see Table 1).

Similarly, no fixed pattern is observed for the factor sampling station in which the communities differ in ordination space (see Figure 3). The lack of a clear separation of community structure across sampling stations can be caused by the relatively close proximity of the sediments used in this study (less than 70 km). In a global survey using a metagenomic approach, geographical distance was demonstrated to be a more important variable in explaining variance between bacterial communities in marine sediments than ecosystem type [28]. Also, lateral transport of sediment material is known to occur in the Baltic Sea [29] and can further reduce the influence of location on the bacterial community structure. Notwithstanding, sampling location explains 6% of variation in the total dataset. This effect is smaller when the influence of cyanobacterial DNA is excluded from the data, but still accounts for 4% of the community structure variance in the exbin72 dataset (see Table 1).

The bacterial communities in slurries that were amended with carbon, nitrogen and phosphorus were not separated from the control communities in ordination space. The factor amendment only explained 3% of the variation in the exbin72 dataset.

Summarizing, for all factors (redox condition, station, sample fraction, and amendment) there is no clear pattern in which the bacterial community structures differ, except for the variance caused by high abundance of cyanobacteria on the basis of bulk DNA at stations LF1 and LF1.5 (see Figure 3).

Additional environmental variables potentially forcing microbial community structure are salinity, pH, light availability, and temperature. The variable typically explaining most variation between microbial communities from environments as diverse as oceans and deserts, is salinity [30]. At our sites, however, bottom water salinity varies over only a limited range from 8 to 12 [7]. No trend is observed in community structure from lower salinity (station LF1) to higher salinity (station LF5; see Figure 3). Thus, salinity cannot explain the differences in community structure between sample locations.

For soil environments the structuring of microbial communities globally is governed by pH [31]. Due to the buffering capacity of seawater, the pH ranges in marine systems usually are not as extreme as in soils, possibly reducing the influence of pH on marine community structure. The in situ pore water pH was similar for the sampled stations (7.1–7.8) [7]. However, the pH range of the slurries at the end of the incubation period was wider (4.9–7.8, median pH = 6.9, see Figure S1). Decrease of pH during incubation occurs mainly as a result of CO_2_ production during organic matter mineralization. The CO_2_ production was on average lower in the anoxic incubations (determined during the first week of the incubation period) [7]. In addition, the alkalinity of the anoxic slurries can increase due to the production of for example sulfide and ammonia during anoxic mineralization, resulting in an increased capacity of the anoxic slurries to withstand lowering of the pH [32]. As a result, the average pH of the oxic slurries is significantly lower than of the anoxic slurries (6.22 and 7.35, respectively, Student’s t-Test, P<0.001; see Figure S1). Although the pH of the incubations is dependent on the redox conditions, the percentage of community structure variance that can be explained by the pH is low (PERMANOVA; 4% for both DNA and RNA fraction, 2% for whole-cell fraction; p = 0.001). Due to the limited incubation volume, the pH effect caused by remineralization is likely overestimated compared to natural sediments (even though the headspace of the incubations was purged weekly). On the other hand, in oxic sediment surface layers the re-oxidation of reduced compounds with oxygen leads to an additional decrease in pH [32].

### Phylogenetic Characterization of Bacterial Communities

The phylogenetic tags assigned to the DGGE bands that were selected for identification are given in Table S3. When it is assumed that DGGE bands at the same position in a gel (bin) consist of the same DNA sequence(s) across lanes, the abundance of bacterial phyla can be determined without the need to identify each band in every lane. However, not all of the 101 DGGE bins contain an identified sequence, either because no DGGE band was excised from a particular bin, or because the sequence of an excised band had a lower than 95% match at phylum level, leaving approximately 40% of the bacterial abundance in this study unidentified.

**Figure 6:**
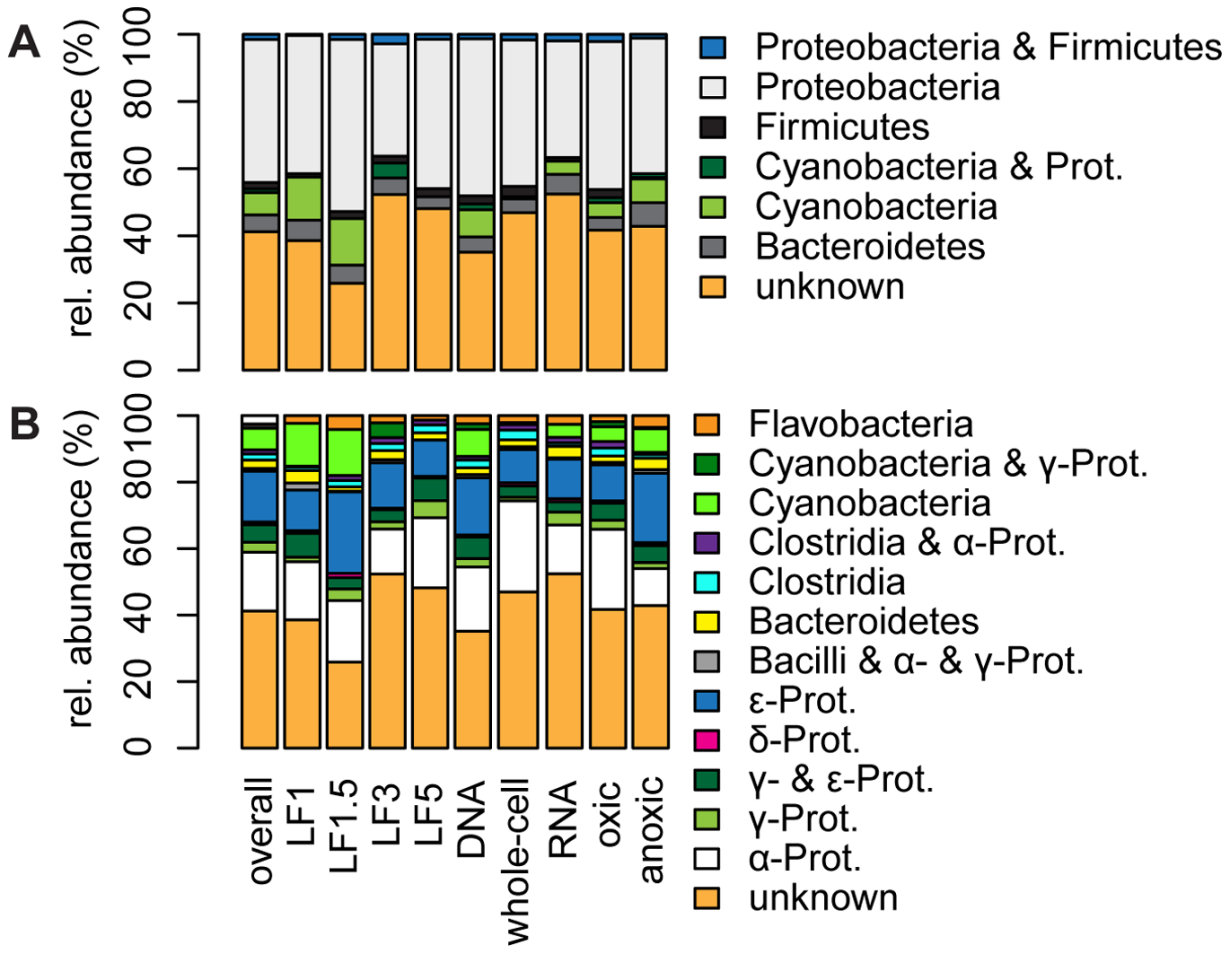
Relative abundance of bacterial phyla (Panel a) and classes (Panel b). The phylogenetic affiliation of selected DGGE bands was determined on the basis of phylogenetic tags assigned by the RDP classifier (see text). DGGE bins from which bands from separate lanes received different tags are classified as such, causing the combined phylogenetic labels of several of the categories. The abbreviation “Prot.” stands for Proteobacteria.

*Proteobacteria* are the most abundant phylum (43% of the intensity over all samples), followed by *Cyanobacteria* (7%), *Bacteroidetes* (5%), and *Firmicutes* (2%; Figure 6a). The high abundance of *Proteobacteria* is in accordance with observations from other global marine benthic bacterial community structure studies [33]. In Baltic Sea sediments, *Proteobacteria* have been found to be either the most abundant phylum [8], or one of the more abundant phyla [9], [22]. *Firmicutes,* whose presence in marine sediments is considered to be related to the proximity of land [28], are present in about 40% of all samples, distributed across stations and incubation treatments. Although this phylum contains many anaerobes, their abundance is similar in oxic and anoxic incubations. In contrast, members of the phylum *Bacteroidetes*, which also contains many anaerobic members, are significantly more abundant in anoxic than in oxic incubations.

When further specified to the class level (Figure 6b and Table S4 in File S1), *Alphaproteobacteria* are the most abundant, closely followed by *Epsilonproteobacteria* (18% and 15%, respectively). Globally, the abundance of *Alphaproteobacteria* is higher in deep sea than in coastal marine sediments [34], [35]. They are generally highly abundant in marine plankton [28], [35], except in anoxic water layers [35]. Also in the present study, *Alphaproteobacteria* are significantly less abundant in the anoxic than in the oxic incubations (11% versus 24% on average; ANOVA Df = 2, F = 9.6026, P<0.001).

In contrast, *Epsilonproteobacteria* are more abundant in the anoxic incubations (21% versus 11%; ANOVA Df = 2, F = 6.1899, P<0.01; see Figure 6b). Members of the *Epsilonproteobacteria* often occur under high-sulfide conditions in marine sediments, and are also the most abundant chemoautotrophic bacteria at the pelagic redoxcline of the Baltic Sea [36]. They are metabolically versatile, capable of both chemoautotrophic sulfide oxidation and heterotrophic metabolism, and are able to utilize a variety of different electron acceptors [37]. The inverse relationship between the abundance of *Alpha*- and *Epsilonproteobacteria* has also been shown in other marine systems, which can be explained in terms of their contrasting ecological preferences [28], [35].

*Gammaproteobacteria* contribute only a minor portion to the bacterial abundance in this study, which is in contrast to the general dominance of *Gammaproteobacteria* in marine benthic communities [28]. Three DGGE bins are classified as belonging to the *Gammaproteobacteria*, however, two of these bins have a mixed classification as bands from this bin in separate lanes are affiliated to different bacterial classes. It is therefore plausible that the abundance of *Gammaproteobacteria* in this study is underestimated by only taking into account the one bin that is classified as solely belonging to the *Gammaproteobacteria*.

Only one DGGE bin (no. 70) was classified as being affiliated to the *Deltaproteobacteria*. The abundance of this bin was the same across incubation treatments and stations. This is surprising, both because *Deltaproteobacteria* can be dominant members of benthic communities [28] and because this class contains many anaerobic members (for example, most of the sulfate-reducing bacteria) and therefore is expected to be more abundant under anoxic conditions.

### Implications for the Benthic P Flux

We did not observe a clear separation between the structures of the active bacterial communities in the oxic and anoxic sediment incubations on the basis of DGGE fingerprinting (see Figures 3 and 5). In addition, the fingerprints of the in situ total bacterial communities of the four sampling stations are similar, while large differences in benthic release of P are observed between these sites (e.g. [10]). We therefore conclude that bacterial community structure in itself has a limited direct impact on benthic fluxes of P in the Baltic Sea.

However, the results do show differences in the relative abundance of individual taxa for contrasting redox conditions, even at the higher taxonomic level that we used to characterize the bacterial communities. How these differences in relative abundance impact P cycling depends on the characteristics of the taxa. For example, the higher relative abundance of *Alphaproteobacteria* under oxic conditions may result in a more efficient uptake of small organophosphates, as marine *Alphaproteobacteria* are over-represented with respect to the number of genes responsible for uptake of glycerol phosphate [38]. In addition, the low abundance of cyanobacterial RNA compared to DNA (see Figure 4) can be the outcome of enhanced P release, caused by preferential degradation of RNA. RNA is a biomolecule that is particularly rich in P compared to bulk marine organic matter (C:P RNA = 9.5∶ 1; marine organic matter ≈ 106∶1) [39], [40]. To gain more insight in the relation between specific members of the bacterial community and P cycling future work should employ a more targeted approach (e.g. at polyphosphate-accumulating bacteria) than the community-level approach of this study. The release of P to pore-water during organic matter mineralization depends on both the P content of the organic matter and the P uptake by microorganisms. The C:P ratio of prokaryotes from the whole-cell fraction of incubations from station LF1 and LF1.5 has been determined by X-Ray Microanalysis in an accompanying study [13]. These prokaryotes have been shown to be poor in P with C:P ratios of approximately 400∶1. This C:P ratio is much higher than the Redfield ratio for marine organic matter of 106∶1 [39]. The C:P ratio of prokaryotes did not differ across incubation treatments (redox condition and amendment), which is in line with the lack of change in bacterial community structure observed in the present study. These results suggest that benthic bacteria in Baltic Sea sediments from contrasting redox conditions have only a limited need for P and contribute to the enhanced release of P from organic matter.

In addition, the community structure of the whole-cell fraction clusters together with total and active bacterial communities in these sediments (see Figure 2 and 3), validating the method of density separation of bacterial cells from sediments that was employed for X-Ray Microanalysis. Also, the relative abundance of bacterial phyla in these Baltic Sea sediments is similar to globally observed benthic bacterial communities. This suggests that high C:P ratios determined for Baltic Sea prokaryotes cannot be explained by an unusual bacterial community. Instead it suggests that high C:P ratios of sediment bacteria may be common in environments where conditions do not favor the formation of polyphosphate [13].

## Conclusions

This study shows that benthic bacterial community structures cannot be described as being typical of oxic or anoxic conditions on the basis of DGGE fingerprinting methods. In addition, the fingerprints of the bacterial community structures of sediments from sites with contrasting benthic P fluxes were highly similar. Bacterial community structure in itself therefore has a limited direct impact on benthic fluxes of P in the Baltic Sea.

The relative contribution of specific members of the communities is consistently different for oxic and anoxic conditions, showing that a more targeted approach than fingerprinting of total bacterial communities (for example using next-generation sequencing) is necessary to trace community differences in response to changing redox conditions. Fingerprinting of microbial community structure at DNA level without sequence information is especially prone to misinterpretation, as shown by the large influence of presumably inactive cyanobacterial DNA on bacterial community structure in this study.

## Supporting Information

Figure S1 Boxplot of pH values of the oxic and anoxic slurries at the end of the incubation period.
(PDF)

Table S3 Overview of 16S rRNA gene sequences, BLASTN matches and phylogenetic tags assigned by the RDP classifier of excised DGGE bands.
(XLSX)

File S1 Contains Table S1 (Sampling station characteristics), Table S2 (DGGE bins with a significant fit to the community structure CA), and Table S4 (Class level abundance of DNA, whole-cell, and RNA fraction of the slurry incubations (%)).
(DOCX)
