# Correction: Effect of Redox Conditions on Bacterial Community Structure in Baltic Sea Sediments with Contrasting Phosphorus Fluxes

**DOI:** 10.1371/journal.pone.0102288

**Published:** 2014-07-02

**Authors:** The PLOS ONE Staff

The following information is missing from the Funding section: “This publication is publication number 5585 of the Netherlands Institute of Ecology (NIOO-KNAW), and publication number DW-2014-1003 of the Darwin Center for Biogeosciences.” The publisher apologizes for this error, which occurred during the preparation of the article for publication.
