# Supplementary figures and images for: Effect of Redox Conditions on Bacterial Community Structure in Baltic Sea Sediments with Contrasting Phosphorus Fluxes
Source: PLoS One. 2014 Mar 25;9(3):e92401. doi: 10.1371/journal.pone.0092401 (PMC3965429; doi:10.1371/journal.pone.0092401)

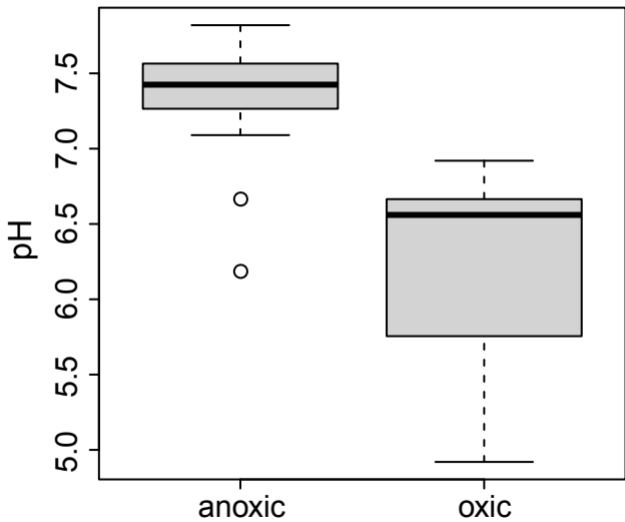

Supplement: Figure S1 — Boxplot of pH values of the oxic and anoxic slurries at the end of the incubation period. (PDF) [file pone.0092401.s001.pdf]
