# Supplementary material for: Effect of Redox Conditions on Bacterial Community Structure in Baltic Sea Sediments with Contrasting Phosphorus Fluxes
Source: PLoS One. 2014 Mar 25;9(3):e92401. doi: 10.1371/journal.pone.0092401 (PMC3965429; doi:10.1371/journal.pone.0092401)
Supplement: File S1 — Contains Table S1 (Sampling station characteristics), Table S2 (DGGE bins with a significant fit to the community structure CA), and Table S4 (Class level abundance of DNA, whole-cell, and RNA fraction of the slurry incubations (%)). (DOCX) [file pone.0092401.s003.docx]

**Supporting Information File S1**

**Table S1:** **Sampling station characteristics**.

| station | LF1 | LF1.5 | LF3 | LF5 |
| --- | --- | --- | --- | --- |
| coordinates | 57º35’22’’N | 57º35’23’’N | 57º35’42’’N | 57º35’32’’N |
|  | 21º10’06’’E | 21º07’28’’E | 20º27’36’’E | 20º03’46’’E |
| O_2_ penetration depth sediment (mm) | 1.5 | < 1 | nd* | nd* |
| H_2_S in bottom water (*µ*mol L^-1^ ) | nd | nd | 2.97 | 21.08 |
| Salinity bottom water | 7.9 | 8.2 | 10.1 | 12 |
| Temperature bottom water | 4 | 4.3 | 5.7 | 6.7 |
| O_2_ in bottom water (mL L^-1^) | 4.8 | 3.8 | 0.7 | 0 |
| Depth (m) | 67 | 72 | 95 | 135 |
| Pore water pH | 7.1 | 7.2 | nd | 7.8 |
| Organic matter content (% dry weight) | 10 | 16 | 20 | 19 |
| Pore water phosphate (*µ*mol L^-1^) | 94.7 | 67.4 | nd | 8.5 |
| Total P (*µ*mol g^-1^ dry weight) | 32 | 40 | 48 | nd |
| P org (*µ*mol g^-1^ dry weight) | 6.4 | 10.4 | 16.4 | nd |
| C_org_ (%) | 2.6 | 5.4 | 9 | nd |
| C_org_:P_org_ | 340 : 1 | 437 : 1 | 466 : 1 | nd |
| C_org_:P_tot_ | 57 : 1 | 116 : 1 | 172 : 1 | nd |
| phosphatase activity (*µ*mol g^-1^ dry weight h^-1^ ) | 0.89 | 1.79 | 1.56 | 1.55 |
| NH4^+^/HPO_4_^2-^ flux ratio (molar)** | 0.08 | 3.31 | 19.94 | nd |

Pore water and sediment data are averaged values for the top centimeter. (nd: not determined; *: sediment oxygen profiles not determined as sulfide was detected in bottom water; **: values from ref [[10](#_ENREF_10)]). Table adapted from ref [[7](#_ENREF_7)].

**Table S2: DGGE bins with a significant fit to the community structure CA.**

| bin | r^2^ | Pr(>r) | phylum |
| --- | --- | --- | --- |
| 72 | 0.58 | *** | Cyanobacteria (3/4) & Proteobacteria (1/4) |
| 101 | 0.29 | *** | Proteobacteria |
| 78 | 0.28 | *** | unknown |
| 108 | 0.24 | *** | unknown |
| 77 | 0.19 | *** | unknown |
| 73 | 0.18 | *** | Proteobacteria |
| 102 | 0.16 | *** | unknown |
| 109 | 0.11 | *** | Proteobacteria |
| 58 | 0.09 | ** | Bacteroidetes |
| 36 | 0.08 | ** | unknown |
| 61 | 0.08 | ** | unknown |
| 90 | 0.08 | ** | unknown |
| 89 | 0.06 | * | Proteobacteria |
| 64 | 0.06 | * | Proteobacteria |
| 53 | 0.05 | * | Bacteroidetes |
| 48 | 0.05 | * | none |
| 60 | 0.05 | * | Bacteroidetes |
| 120 | 0.05 | * | none |
| 55 | 0.05 | * | none |
| 88 | 0.05 | * | unknown |
| 92 | 0.04 | * | unknown |
| 91 | 0.04 | * | Proteobacteria(2/3) / Firmicutes (1/3) |

Significance codes: *** = 0.001, ** = 0.001 < 0.01, * = 0.01 < 0.05. DGGE bins that do not contain a band with a phylogenetic tag are labeled “unknown”.

**Table S4: Class level abundance of DNA, whole-cell, and RNA fraction of the slurry incubations (%).**

| class affiliation | fraction | total | oxic | anoxic | control | CNP |
| --- | --- | --- | --- | --- | --- | --- |
| unknown | DNA | 28 | 27 | 28 | 23 | 32 |
|  | whole-cell | 47 | 49 | 45 | 40 | 52 |
|  | RNA | 53 | 50 | 56 | 51 | 55 |
| Alphaproteobacteria | DNA | 14 | 23 | 5 | 18 | 10 |
|  | whole-cell | 27 | 34 | 21 | 34 | 22 |
|  | RNA | 13 | 17 | 10 | 14 | 13 |
| Gammaproteobacteria | DNA | 2 | 4 | 1 | 3 | 2 |
|  | whole-cell | 1 | 2 | 0 | 1 | 1 |
|  | RNA | 3 | 3 | 4 | 3 | 3 |
| Gamma- & Epsilonproteobacteria | DNA | 8 | 8 | 9 | 7 | 10 |
|  | whole-cell | 3 | 2 | 5 | 2 | 5 |
|  | RNA | 3 | 5 | 2 | 4 | 2 |
| Deltaproteobacteria | DNA | 0 | 0 | 0 | 0 | 0 |
|  | whole-cell | 1 | 1 | 1 | 1 | 1 |
|  | RNA | 1 | 1 | 2 | 1 | 1 |
| Epsilonproteobacteria | DNA | 24 | 19 | 30 | 26 | 22 |
|  | whole-cell | 10 | 3 | 18 | 10 | 10 |
|  | RNA | 12 | 10 | 14 | 11 | 13 |
| Bacilli & Alpha- & Gammaproteobacteria | DNA | 1 | 1 | 2 | 0 | 2 |
|  | whole-cell | 1 | 1 | 0 | 0 | 1 |
|  | RNA | 1 | 0 | 1 | 0 | 1 |
| Bacteroidetes | DNA | 2 | 1 | 3 | 0 | 5 |
|  | whole-cell | 2 | 0 | 4 | 1 | 3 |
|  | RNA | 3 | 4 | 3 | 4 | 3 |
| Clostridia | DNA | 1 | 2 | 1 | 1 | 1 |
|  | whole-cell | 3 | 4 | 2 | 2 | 3 |
|  | RNA | 1 | 2 | 1 | 1 | 1 |
| Clostridia & Alphaproteobacteria | DNA | 1 | 0 | 1 | 1 | 1 |
|  | whole-cell | 2 | 2 | 1 | 3 | 1 |
|  | RNA | 2 | 3 | 0 | 3 | 0 |
| Cyanobacteria | DNA | 12 | 9 | 16 | 16 | 9 |
|  | whole-cell | 0 | 0 | 0 | 0 | 0 |
|  | RNA | 4 | 4 | 4 | 5 | 3 |
| Cyanobacteria & Gammaproteobacteria | DNA | 2 | 4 | 1 | 3 | 2 |
|  | whole-cell | 1 | 0 | 1 | 1 | 0 |
|  | RNA | 0 | 0 | 0 | 0 | 0 |
| Flavobacteria | DNA | 3 | 2 | 4 | 2 | 4 |
|  | whole-cell | 2 | 1 | 3 | 4 | 1 |
|  | RNA | 3 | 2 | 3 | 2 | 4 |
